# Nutritional Characterization of a Traditional Cultivar of Tomato Grown Under Organic Conditions—cv. “Malacara”

**DOI:** 10.3389/fnut.2021.810812

**Published:** 2022-01-11

**Authors:** María D. Raigón, María D. García-Martínez, Octavian P. Chiriac

**Affiliations:** ^1^Instituto de Conservación y Mejora de la Agrobiodiversidad Valenciana/Departamento de Química, Universitat Politècnica de València, Valencia, Spain; ^2^Department of Agricultural, Forest and Food Sciences, University of Turin, Turin, Italy

**Keywords:** biodiversity, flavors of the past, for hanging, red exocarp, yellow exocarp

## Abstract

The loss of genetic diversity due to the replacement of local tomato (*Solanum lycopersicum* L.) varieties by improved cultivars has been mitigated in many cases by the good work of organic farmers in maintaining local agricultural biodiversity. In parallel to these initiatives, in recent years, consumers have developed an increasing awareness of both food-related health, environmental issues, and food demand to recover the flavors of the past. In the case of tomatoes, these attributes (nutritional, organoleptic, social, and environmental) are closely related to organic production using local varieties. “Malacara” tomato is an example of a local variety. Coming from Sierra de Cádiz, it is a varietal type called “Cuelga” (“for hanging,” because the tomato trusses are hung from beams in the farmhouses). Cultivated and harvested in the open air during the summer months, these tomatoes are commercialized and consumed in the winter. Historically, this variety has enabled the fresh consumption of tomatoes during the winter, without the need to force cultivation. It is highly appreciated in the local cuisine and is the basis for sauces figuring in typical dishes. Its characteristic traits are small, pallid fruits, and long shelf life. The main objective of this work has been to typify two Malacara tomato cultivars (red and yellow color) grown under organic farming conditions, through the characterization of morphological, nutritional, and volatile parameters. The main differences are due to morphological parameters (fruit weight and color of the exocarp and endocarp). Other characteristics such as the content of ash, fiber, moisture, the concentration of iron, magnesium, and calcium, and content of lycopene are different between both cultivars. This study provides information on the nutritional and aromatic composition of two Malacara tomato cultivars, differentiated by their color and grown under organic farming conditions. The results add value to the native horticultural heritage and can aid in the selection of tomato varieties suitable for a sustainable production system and to produce tomatoes with high nutritional value and rich in aroma.

## Introduction

Plant genetic resources for food and agriculture, and specifically, traditional varieties, play an important role in the sustainability and security of the global food system. Traditional varieties are an essential component of agricultural biodiversity, guaranteeing agricultural production adapted to the territory and ensuring the livelihood of a large proportion of people who depend on agriculture (1). Genetic diversity within crop species is wide. Germplasm grown under local environmental conditions can be optimized for small regional production areas that adjust to prevailing environmental and climatic patterns. However, in recent years there has been the phenomenon of genetic erosion within species, that is, “the loss of individual genes and the loss of particular combinations of genes, such as those manifested in locally adapted varieties” (2). This erosion has been supported for the contemporary plants breeding investigations, more focused on increasing the productivity of some crop species, than on enhancing cultivated genetic diversity (3). The work that small farmers have traditionally carried out in the conservation of genetic material adapted to local conditions (soil, climate, and consumption) must be highlighted, and in particular, the important role of organic farmers (4).

The health of people and the planet are at critical moments. There are synergies between intensivist global food systems and phenomena such as climate change, malnutrition, and obesity. Coexisting with scenarios of global loss of biodiversity, instability in the planet's natural systems, limits of the phosphorus and nitrogen cycles, lack of water resources, along with social and economic disturbances (5). The alternatives to these issues go through the development of a sustainable food system, including organically grown food. And consequently, the area devoted to the organic crop increases, ~2% annually worldwide (6). In 2019, Spain was the third country in the world with the largest organic area, behind Australia and Argentina. Organic horticultural production, in Spain, is in the fifth position, behind cereal, olive tree, dried fruit, vineyard, and legume acreage. In line with this trend, an increasing proportion of tomato (*Solanum lycopersicum* L.) production is organically grown. The continuous growth in Spain of the agricultural area under organic production responds to the marked increase in organic consumption in European markets, and also, the growing demand in the domestic market. The main commercialization channels for autochthonous varieties are the local markets, better adapted to specific agro-climatic conditions, and especially recommended for organic agriculture. This phenomenon is observed with greater intensity in exquisite crops such as tomatoes (7, 8).

Tomato is an excellent source of nutrients and bioactive antioxidant compounds that are important for human health, including minerals, vitamins C and E, β-carotene, lycopene, flavonoids, organic acids, phenolics, and chlorophyll (9, 10). Some of the tomato components mentioned above have antioxidant properties (11), while others, such as sodium, potassium, magnesium, calcium, manganese, copper, zinc, and iodine, may reduce the risk of cardiovascular diseases (12) and its organic acids that may contribute to maintaining acid-base balance (13). The chemical composition of the tomato fruit depends on factors such as crop system, fruit maturity, environmental conditions (soil and climate), and the cultivation method in which the plants are grown (14–16). The results regarding the research on the effects of organic and conventional production on tomato quality are sometimes contradictory. In terms of quality, some studies report better taste, higher vitamin C contents, and higher levels of other quality-related compounds for organically grown tomatoes (17–19).

The identification of cultivars with high nutritive value represents a useful approach to selecting tomato cultivars with better quality and health-promoting properties. The diversity of tomatoes rapidly declined during the 20th century as a result of the industrialization of agriculture and the advance of plant breeding programs. Part of this diversity was collected and conserved in germplasm banks. The Spanish National Inventory contains 2,634 accessions of tomato conserved in different Spanish institutions. Several efforts have been made to characterize Spanish tomato materials (20, 21), there are even works on the characterization of tomato cultivars under organic farming conditions (22) and works that have characterized some varieties of tomato called “Cuelga” (23) or “for hanging” because the tomato trusses are hung from beams in the farmhouses. These types of tomatoes are grown and harvested during the natural cycle, in the open air during the warm months, and are commercialized and consumed in the winter. Historically, these various types have enabled the fresh consumption of tomatoes during the winter, without the need to force cultivation in greenhouse conditions. “Cuelga” tomatoes are a varietal type, which comprises a set of cultivars (integrated by inbreds) with great heterogeneity in fruit morphology. It is highly appreciated in the local cuisine and is the basis for sauces figuring in typical dishes. The characteristic traits are small, pallid fruits and long shelf life. In this context, this study mainly aims to characterize two Malacara tomato cultivars, differentiated by the red and yellow exocarp color, grown under organic farming conditions, through the characterization of physical parameters and nutritional composition. The literature shows the typification of other “Cuelga” tomatoes, but it is the first time that the nutritional composition of Malacara tomatoes has been characterized, identifying characteristics between the fruits of the two cultivars of different colors.

## Materials and Methods

### Plant Material

Tomato crop was carried out during the summer in 2019 in the “La Verde” cooperative in Villamartín (Cadiz, Spain) (36° 52′ 0″ N, 5° 38′ 0″ W). The cooperative has been involved with organic agriculture certificates for 33 years. Its agricultural activity is mainly directed toward horticultural production. The cooperative has three hectares to produce nectarines, apples, pears, plums, citrus, and figs, and one hectare of olive grove, which is also needed to increase biodiversity. The rest of the area (ten hectares) is divided into 14 plots for the cultivation of horticulture species, which vary according to the season, following a precise crop rotation. The cooperative promotes re-seeding and seed exchange, understood as a model of *ex situ* conservation in the country, involving the maintenance of varieties by their cultivation and closing cycles. At present, its seed bank contains over 250 varieties available in some 40 different species (24), including among the traditional varieties, the Malacara tomato seeds, which they grow for the local market. Although the local varieties have a high genotypic heterogeneity, the Malacara tomato has been in the “La Verde cooperative” for ~25 years ago, as a result of seed exchange between local producers. It is possible that the lack of specialized markets has caused this tomato to spread little by other farmers. But the specific conditions of the cooperative (strong commitment to indigenous seeds, conservative tradition, local markets, and diversification) have made it possible to maintain these cultivars and introduce them annually on the market. Therefore, although the origin of the samples is from a single source, it is very robust to achieve the objectives.

The Malacara tomato was grown outdoors in the 2019 harvest, following organic farming methods, in clay-loam soil, fertilized with sheep manure in quantities of 2 kg of manure per m^2^ and year. Plantlets were raised in seedling trays filled with organic compost and kept in an insect-free climate chamber until transplanted. No fertilization or phytosanitary treatments were applied before the transplant. Plants were cultivated in the summer cycle. The seedbed was carried out in February and the transplant to the field was carried out in April. A month later, the training of the plants is done. 1,000 tomato plants were grown, of different varieties, and 200 plants were from Malacara (100 for each cultivar and plot). The plants were spaced 1.2 m between rows and 0.45 m within the row. A total of 8 L of water per plant and week, in the pre-fruiting phase, were applied by a drip irrigation system, decreasing the frequency after fruiting. The average temperatures for the period were 15°C minimum and 30°C maximum. Weeding was done manually between plants, with three applications throughout the growing cycle, and mechanically between lines, with one application. Phytosanitary treatments consisted of the authorized use of sulfur powder as a preventive treatment. Harvesting of the tomato fruits began in June and continued until the end of October. In both cultivars, common practices of transplanting, weeding, training, pruning, and harvesting were similar.

**Figure 1:**
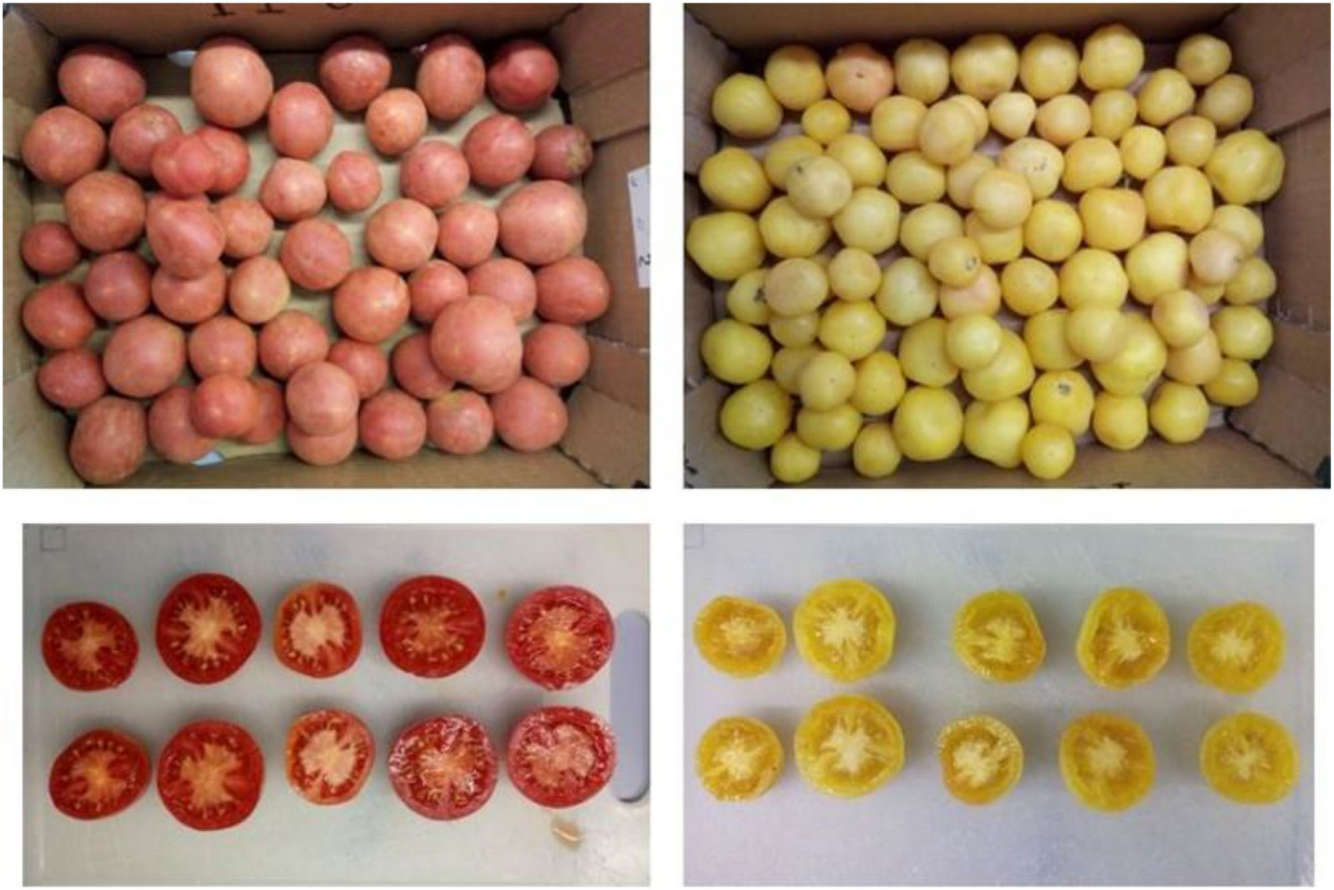
Red and yellow type of tomatoes cv. Malacara.

Samples of tomatoes are shown in Figure 1. Tomato fruits were collected homogeneously, by hand, selecting fruits from the central plants of the plot. The fruits come from two harvests made commercially ripe, in early September. Of the collected fruits, ~100 sample fruits of Malacara tomatoes by each color were supplied for analysis.

For composition determinations, the fruits were transversely cut in half, and the seeds were eliminated. One-half of the tomatoes were grouped and squeezed with a domestic extractor for the analysis of pH, titratable acidity, contents in soluble solids, reducing sugars (glucose, fructose), vitamin C, carotenoids, total polyphenols, and total antioxidant capacity analysis. The other tomato halves were homogenized, and one aliquot was dried for the proximate and mineral determination. Another fraction of fresh tomato was immediately freezing, in a vial and hermetically closed, for subsequent determination of volatile components. When using a headspace chromatography methodology, the volatile components are not significantly altered.

### Morphological Parameters

The morphological parameters such as tomato dimensions (unit weight, smaller diameter, larger diameter, smaller height, larger height) and color parameters of exocarp, mesocarp, and endocarp were monitored on 60 tomatoes by cultivar. The fruit weights were measured with an analytical balance (CB-Junior, Cobos) with an accuracy of ± 0.01 g. The fruit's dimensions were measured using an electronic digital slide gauge (model CD-15 DC; Mitutoyo (UK) Ltd, Telford, UK) to within 0.01 mm accuracy. Color measurement in CieLab space (25) was carried out using a colorimeter (Konica Minolta CR-300, Photo Imaging Inc., Mahwah, NJ, USA). In each measurement the values of the three coordinates (L^*^, a^*^, b^*^) are obtained which, combined with each other, gives rise to the color index. The Chroma (C) of each fruit is calculated using the formula: $C=\;\sqrt{a^{2}+b^{2}}$

The Hue-Angle (H) of each fruit is calculated using the formula: $H=\;\tan^{-1}\left(\frac{b}{a}\right)$

The color index (CI) of each fruit is calculated using the formula: $CI=\;\frac{1000^{*}a}{L^{*}b}$

The C shows the greater or lesser saturation toward that certain color. A high value of C is a highly saturated color. A value of zero for C indicates an achromatic stimulus. Hue (H) is the property of color associated with the dominant wavelength. With CI values close to 0, the yellow tones are evaluated, and values close to 20, the red tones are evaluated (26).

### Nutritional Parameters

Proximate composition was carried out by official methods: moisture (AOAC 984.25), proteins (AOAC 984.13), fat (AOAC 983.23), fiber (AOAC 991.43), and ashes (AOAC 923.03). The carbohydrate (CH) content was calculated by difference. The energy was calculated by multiplying by 9 kcal the grams of fat and 4 kcal the grams of protein and carbohydrates each 100 g of tomato. The final results are expressed as g·100 g^−1^ of fresh weight (fw).

The mineral composition was determined by previous digestion of the samples to the method Association of Official Analytical Chemists (AOAC) 985.35. The samples were calcined in a Carbolite CWF 1100 muffle at 550°C, the ashes were dissolved and settled with concentrated HCl until a 2% HCl solution. The calibration curves were made by diluting the standards to the specific concentrations for each element. The analytical curves were obtained with a linear response for the selected concentration ranges. Mineral analysis was performed by atomic absorption spectroscopy, in a Thermo elemental AAseries spectrometer, software v.11.03, and hollow cathode lamps for each element, except for the phosphor, which was analyzed by colorimetry (27) using a UviLine 9400 (Schott Instruments) spectrometer. The determination of the soluble solids content (SSC) present in the tomato juice was carried out by refractometric techniques (27). The material used in this determination is in a hand-held refractometer with a range of 0–32 °Brix. The pH determination was made by direct potentiometric measurement of the homogenized tomato juice with pH & Ion-metro GLP 22 (CRISON). The determination of the total acidity (TA) consists of the potentiometric titration of the sample with an alkaline solution (0.5 *N* NaOH) up to pH = 8.1 of the acidity of the tomato juice (27). The results are expressed in grams of citric acid for 100 g of sample. The taste index of tomato (S), expressed in percentage, is based on the soluble solids content and total acidity content of fruit (28) and is determined by the expression: $S=TA+\frac{SSC}{20^{*}TA}$. The optimal value for flavor balance is considered when the taste index is higher than 1.

Contents in reducing sugars (fructose and glucose, F+G) were determined by titration with thiosulfate (27). The vitamin C (Vit C) concentration in the tomato juice was determined by potentiometric titration with chloramine-T, using an automatic titration equipment (702 SM Titrino, Metrohm, Herisau, Switzerland), the results were expressed as mg 100 g^−1^ fw of tomato. A UviLine 9400 (Schott Instruments) spectrometer was used to measure the absorbance for carotenoids, polyphenols, and antioxidants. The carotenoids, lycopene (Ly) and β-carotene (β-car), were determined by extraction in darkness with ethanol:hexane (4:3 v/v) and UV/V spectrophotometry following the protocol developed by Zscheille and Porter (29) with the modification of Russeaux et al. (30). For the total carotenoids (TC), the absorbance of the samples at 452 and 510 nm wavelengths were measured. Results were expressed as μg 100 g^−1^ fw. Total polyphenols (TP) were determined in an aliquot of the methanolic extract by a modification of the Folin–Ciocalteu assay, according to a previously published protocol (31), using gallic acid as the reference standard. The results are expressed in mg of gallic acid for 100 g of fruit fresh (mg EAG 100 g^−1^ fw). To measure the effect of the extract on the DPPH (2,4-dinitrophenylhydrazine) radical, the optimized method of Brand-Williams (32) adapted to tomatoes was used. This measure of the total antioxidant (AOT) capacity, it is carried employing methanol:HCl (99:1) as a dissolvent. The results are expressed in an activity equivalent to Trolox (μmol 100 g^−1^ of fresh fruit weight).

### Volatile Composition

Headspace/solid-phase microextraction (HS/SPME) was used for the extractions of the volatile fraction according to the methodology of Moreno et al. (33). For the analysis, 2 g of fruit mesocarp per sample were put into a 20 mL sealed headspace vial, which was pre-incubated at 40°C for 30 min. After that, 40 min of extraction at 40°C was carried out, using an SPME fiber Divinylbenzene/Carboxen/Polydimethylsiloxane (DVB/CAR/PDMS, 50/30 μm) (Supelco, Bellefonte, PA, USA) which was introduced into the vial headspace and adsorbed the volatiles. Then, the thermal desorption of the fiber was performed in the splitless mode at 250°C for 30 s in the gas chromatograph injection port. The analysis of volatiles was performed using a 6890 N Network Agilent gas chromatograph coupled by a Life-T-effluent (1:1) to a 5973 Inert Mass Selective detector (Agilent Technologies, Santa Clara, CA, USA). A silica capillary column (stationary phase: 5% phenyl, 95% dimethylpolysiloxane; 30 m × 0.25 mm × 0.25 μm) was used and helium was employed as carrier gas (1 mL·min^−1^). MSD ChemStation D.02.00.275 (Agilent Technologies) was used to perform the chromatograms and mass spectra. For the identification of volatile compounds, a comparison of their mass spectra and GC retention time with reference commercial standards were carried out (RS. Sigma-Aldrich, Saint Louis, MO, USA), or tentatively by comparison of the mass spectrum (MS) with the NIST Mass Spectral library (MS Search 2.0) according to other bibliographic data or data from our own research library. For quantification, a total ion current chromatogram (TIC) was employed to integrate the peak area of each compound similarly to other previous works (34). Total volatiles and each individual volatile was studied on the basis of the GC-peak areas (traces values were not considered).

### Statistical Analysis

Every parameter analyzed was done in triplicate. Data obtained were processed using Statgraphics Plus version 5.1 software (StatPoint Technologies, Warrenton, VA, USA) which computed the means and standard errors. Differences between the effects of type, red (R) and yellow (Y) were tested using an ANOVA one-way analysis, and differences among groups were identified with the F-test and the Kruskal-Wallis test which compares medians instead of means at the 95.0% confidence level. The Kruskal-Wallis test tests the null hypothesis that the medians of parameters within each of the two types of Malacara tomato are the same. And it requires the fulfillment that the populations are normal, independent, and present homoscedasticity. Pearson's linear coefficients of correlation (r) between traits were calculated (*n* = 6) from regression analyzes between pairs of traits.

## Results

### Morphological Parameters

Table 1 shows the results of the morphological parameters in Malacara tomato's fruits red and yellow. Statistically highly significant differences were found between all the morphological parameters (size and color) between all the two types of Malacara tomatoes studied. The red type was characterized for more variability for the morphological parameter than the yellow type. Red-type tomatoes are larger in size. On average the differences in weight are ~22.5 g, the average differences in diameter are 7.2 mm, and the differences in height are 6.87 mm, in favor of red type Malacara tomatoes. The shape of the fruits is round, the width/length ratio is close to 1 (1.09 for yellow type and 1.03 for red type). For the color, in the L^*^ it is seen that the exocarp of tomatoes present a measurement difference of 7.29, which means that the yellow Malacara tomatoes are significantly lighter than those of the red type. Also, the mesocarp and endocarp of the yellow-type tomatoes are more luminous, with differences of ΔL = 8.94. The a^*^ plane value for the exocarp, meso, and endocarp of tomatoes is significantly higher for Malacara red-type tomatoes, confirming visual appreciation. The significant differences between the values of plane b^*^, in the different parts of the tomato, also confirm the appreciation of the yellow color. The results show that the chroma, regardless of the part of the fruit, of the yellow Malacara tomatoes is significantly higher, that is yellow color is more marked than red. The tonality of the tomatoes is significantly higher, both in the exocarp, as well as in the meso and endocarp of the red Malacara tomatoes. The CI value close to 15 obtained for the peel and pulp in red Malacara tomato indicate a light shade of red or pink.

**Table 1:** Morphological parameters for the two types of Malacara tomatoes (mean ± SD, *n* = 30 and *p*-value).

**Parameter**	**Malacara Tomato**		***P*-value**
	**Yellow**	**Red**	
Fruit weight (g)	30.77 ± 10.18	53.23 ± 16.69	0.0000
Fruit width (mm)	38.35 ± 4.99	45.55 ± 4.84	0.0000
Fruit length (mm)	35.17 ± 3.65	42.04 ± 5.25	0.0000
L* exocarp	70.86 ± 2.05	63.57 ± 2.17	0.0000
a* exocarp	0.80 ± 0.50	15.11 ± 1.79	0.0000
b* exocarp	27.20 ± 2.36	15.68 ± 1.66	0.0000
C exocarp	27.21 ± 2.34	21.83 ± 1.95	0.0000
He exocarp	0.03 ± 0.02	0.77 ± 0.07	0.0000
CI exocarp	0.44 ± 0.32	15.32 ± 2.28	0.0000
L* mesocarp and endocarp	67.95 ± 2.65	59.01 ± 2.02	0.0000
a* mesocarp and endocarp	0.93 ± 0.64	14.98 ± 2.34	0.0000
b* mesocarp and endocarp	26.63 ± 3.08	16.87 ± 1.75	0.0000
C mesocarp and endocarp	26.65 ± 3.06	22.61 ± 2.44	0.0000
H mesocarp and endocarp	0.04 ± 0.03	0.72 ± 0.07	0.0000
CI mesocarp and endocarp	0.53 ± 0.41	15.13 ± 2.28	0.0000

### Nutritional Parameters

The nutritional fraction, proximate composition, and the content of each mineral studied, expressed in 100 g of the red and yellow, fresh Malacara tomatoes shown in Table 2. The total energy for each 100 g of fresh tomato is 46 kcal for yellow type and 36 kcal for red type. This greatest value is due to the high carbohydrates content (9.65 g 100 g^−1^ for yellow tomato and 7.16 g 100 g^−1^ for red tomato) and the lower moisture content of the yellow Malacara tomatoes since the calories from fat in the yellow tomato is 4.31% and in the case of red tomato 6.29%. Significant differences were found between the two types of tomatoes for, energy value, moisture, carbohydrates contain, and total fiber. No significant differences were found between the two types of tomatoes for protein and total fat. The most abundant mineral element in the Malacara tomato, regardless of color, is potassium, followed by sodium and magnesium for the yellow type, compared to phosphorus for red fruits, in third place. The most abundant trace element in the yellow tomato is iron, followed by zinc, and in the red tomato, zinc predominates over iron. Significant differences were found between the two types of tomatoes for calcium, iron, and magnesium content.

**Table 2:** Nutritional fraction and individual mineral content (100 g of fresh fruits) for the two types of Malacara tomatoes (mean ± SD, *n* = 3 and *p*-value).

**Parameter**	**Malacara Tomato**		***P*-value**
	**Yellow**	**Red**	
Total energy (kcal)	45.96 ± 5.05	35.74 ± 1.80	0.0299
Moisture (g)	85.65 ± 1.15	89.26 ± 0.54	0.0081
Protein (g)	1.33 ± 0.18	1.20 ± 0.15	0.3951
Total fat (g)	0.22 ± 0.05	0.25 ± 0.03	0.3530
Fiber (g)	1.84 ± 0.13	1.03 ± 0.03	0.0005
Carbohydrates (g)	9.65 ± 0.98	7.16 ± 0.35	0.0144
Calcium (mg)	16.05 ± 0.49	13.01 ± 0.74	0.0041
Copper (mg)	0.108 ± 0.015	0.117 ± 0.008	0.5185
Iron (mg)	0.459 ± 0.027	0.339 ± 0.060	0.0349
Magnesium (mg)	31.41 ± 3.48	24.43 ± 1.48	0.0330
Phosphorus (mg)	27.04 ± 1.72	27.80 ± 2.49	0.6932
Potassium (mg)	833.59 ± 116.32	768.74 ± 23.13	0.3972
Sodium (mg)	55.68 ± 16.31	52.02 ± 13.73	0.7807
Zinc (mg)	0.351 ± 0.012	0.342 ± 0.030	0.6530

Table 3 shows the average content for the fruit traits evaluated related to tomato flavor and other bioactive characters, of the red and yellow Malacara tomatoes. The traits evaluated in the tomato Malacara demonstrate the importance of its consumption for aspects related to health and sensory attributes. The pH and the soluble solids content in fresh tomato are greatest value for yellow type (pH = 4.20 in yellow vs. pH = 4.14 in red Malacara tomato) and (6.20 °Brix in yellow vs. 5.87 °Brix in red Malacara tomato). The higher values of these parameters in the yellow tomato have, as a consequence, a higher value of the taste index. When considering the traits related to tomato flavor and other bioactive characters, no significant differences (*p* > 0.05) were found among the two types, except for lycopene, total carotenoids, and total antioxidant capacity (Table 3). The values of the parameters of bioactive components in the Malacara tomato are higher for the red type. For vitamin C, although the differences are not significant (*p* = 0.1029), the average contents are 12.4% higher (353.33 mg 100 g^−1^ fw in yellow and 403.33 mg 100 g^−1^ fw in red). Despite the existence of significant differences among means for the two types of Malacara tomato, for the flavor and other bioactive character traits, considerable variation was found within each of the types. In this respect, in most cases the ranges of variation overlap with the other value, the exception being the total antioxidant capacity, showing significant differences between yellow and red Malacara tomatoes, with slight increases for red tomatoes.

**Table 3:** Fruit traits related to tomato flavor and other bioactive characters for the two types of Malacara tomatoes (mean ± SD, *n* = 3 and *p*-value).

**Parameter**	**Malacara Tomato**		***P*-value**
	**Yellow**	**Red**	
pH	4.20 ± 0.10	4.14 ± 0.110	0.5018
Soluble solids content (°Brix)	6.20 ± 0.20	5.87 ± 0.23	0.1318
Total sugar content (g 100 g^−1^ fw)	4.24 ± 0.57	4.25 ± 1.15	0.9865
Total acidity (g citric 100 g^−1^ fw)	0.43 ± 0.04	0.45 ± 0.06	0.7060
Taste index	1.153 ± 0.05	1.111 ± 0.05	0.3477
Lycopene (μg 100 g^−1^ fw)	34.92 ± 6.93	5512.22 ± 1046.29	0.0008
β-carotene (μg 100 g^−1^ fw)	533.85 ± 118.25	585.74 ± 125.58	0.6305
Total carotenoids (mg 100 g^−1^ fw)	0.44 ± 0.09	9.35 ± 1.81	0.0010
Vitamin C (mg 100 g^−1^ fw)	353.33 ± 7.02	403.33 ± 40.50	0.1029
Antioxidant capacity (AOT μmol ET 100 g^−1^ fw)	1238.87 ± 9.49	1284.86 ± 12.40	0.0070
Total phenolic content (mg EAG 100 g^−1^ fw)	55.60 ± 6.83	61.22 ± 17.91	0.6385

### Volatile Composition

A total of 42 volatile compounds were found among the two types of Malacara tomato, grouped in 12 chemical families with quantitative and qualitative differences. Table 4 shows the list of volatile compounds names, retention index (RI), identification method (RS: reference commercial standard, MS: comparison of the mass spectrum with NIST library and bibliographic data), aroma, and GC peak area total mean values. There are some volatile components that have only been found in the yellow tomato, specifically an aldehyde [(E)-2-hexenal)], three esters (methyl octanoate, isopentyl 3-methylbutanoate and methyl dihydrojasmonate), and two terpenes (carvone, α-thujene). Other components have only been detected in the red Malacara tomato, such as a monoterpene (perillene), four carotenoid components (β-cyclocitral, neral or β-citral, geranial or α-citral, and geranylacetone), and two polyphenolic derivatives (guaiacol and eugenol). In addition to the differences by compounds not detected between both types of tomato, statistically significant differences have been found for four monoterpenes (p-cymene, limonene, β-ocimene, and γ-terpinene), for the carotenoids 6-methyl-5-hepten- 2-one, for the phenol derivatives methyl salicylate, and the three nitrogen and sulfur compounds (2-Isobutylthiazole, pentane, 1-nitro and benzyl nitrile), in all cases, except for methyl salicylate, the concentrations are higher in yellow Malacara tomato.

**Table 4:** List of volatile compounds names, retention index (RI), identification method (RS, reference commercial standard; MS, comparison of the mass spectrum with NIST library and bibliographic data), aroma, mean ± SD, *n* = 3 and *p*-value, for the two types of Malacara tomatoes.

**Chemical family**	**Volatile compound**	**RI**	**IM**	**Aroma**	**Malacara Tomato**		***P*-value**
					**Yellow**	**Red**	
Alcohols	2-phenylethanol	1,136	RS	Floral, sweet, honey	489,694 ± 246,807	575,642 ± 238,376	0.6868
Aldehydes	(E)-2-pentenal	718	MS	Green, tomato, orange	252,609 ± 297,412	46,941 ± 27,394.7	0.2989
	Hexanal	806	RS	Green, fresh, fatty, fruity	1.57E6 ± 1.29E6	1.41E6 ± 595,659	0.8562
	(E)-2-hexenal	814	MS	Green, sweet, bitter, fruity	764,938	Not detected	–
	(Z)-2-heptenal	913	MS	Sweet, green, slightly fatty	512,567 ± 90,361	361,269 ± 44,734.7	0.0601
	Benzaldehyde	982	MS	Bitter almond-like	490,981 ± 265,934	313,506 ± 46,386.9	0.4397
	(E,E)-2,4-heptadienal	921	MS	Sweet, creamy, fatty, citrus	255,776 ± 33,903.6	271,685 ± 38,853.4	0.6583
	2-phenylacetaldehyde	1,081	MS	Green, sweet, floral	648,893 ± 309,124	423,507 ± 89,267	0.2918
	Nonanal	1,104	RS	Aldehydic, waxy, rose	785,860 ± 310,322	804,128 ± 200,381	0.9359
	Decanal	1,204	RS	Aldehydic, sweet, waxy	372,449 ± 271,639	98,519.7 ± 18,316.5	0.1563
	(E,E)-2,4-decadienal	1,220	MS	Fatty, sweet, fresh	1.31E6 ± 864,940	595,208 ± 91,270.2	0.2257
Aliphatic and aromatic hydrocarbons	Styrene	883	MS	Almond	213,567 ± 60,656	104,270 ± 53,238.7	0.0789
	α-methylnaphthalene	1,345	MS	Green musty	123,585 ± 49,054.7	81,914.5 ± 27,784.3	0.3677
	Biphenyl	1,367	MS	Bergamot, cinnamon	143,821 ± 91,346.7	70,658 ± 43,686.8	0.2789
Esters	Methyl octanoate	1,083	MS	Green, fruity, waxy, citrus	155,334.33	Not detected	–
	Isopentyl 3-methylbutanoate	1,054	MS	Fruity, sweet, green	286,361.5	Not detected	–
	Methyl dihydrojasmonate	1,657	MS	Floral, citrus	177,300	Not detected	–
Furans	2-pentylfuran	1,040	RS	Fruity, green, earthy	2.31E6 ± 750,558	2.54E6 ± 740,288	0.7284
Ketones and methylketones	1-octen-3-one	943	MS	Earthy, fungal, green, oily	260,005 ± 26,349.7	248,907 ± 151,929	0.9068
	acetophenone	1,029	RS	Floral, sweet, pungent	58,117.3 ± 7,932.4	64,646 ± 41,419.5	0.7916
Monoterpenes	α-thujene	902	MS	Herbal	55,256.67	Not detected	–
	p-cymene	1,042	MS	Sweet, soft, fresh, lemon	5.59E6 ± 813,109	3.06E6 ± 1.46E6	0.0591
	Limonene	1,018	RS	Citrus, herbal, terpenic	1.56E6 ± 182,424	726,484 ± 355,297	0.0220
	β-ocimene	958	RS	Floral, sweet, herbal, warm	517,027 ± 50,384	182,952 ± 77,229.3	0.0033
	γ-terpinene	998	MS	Citric, fatty, terpenic	4.38E6 ± 523,156	2.46E6 ± 250,811	0.0104
	Perillene	1,125	MS	Flowery, citrus-like	Not detected	570,774	–
Terpenes	Carvone	1,190	MS	Spearmint	70,377.33	Not detected	–
	Camphor	1,121	MS	Hot Turkish spices	130,320 ± 33,954.6	121,242 ± 93,096.3	0.9088
Carotenoids	6-methyl-5-hepten-2-one	938	MS	Lemon-grass	161,207 ± 29,513.3	1.01E7 ± 998,980	0.0001
	β-cyclocitral	1,204	MS	Tropical, herbal, sweet	Not detected	167,245.33	–
	Neral (β-citral)	1,174	MS	Lemon	Not detected	186,223.33	–
	Geranial (α-citral)	1,174	MS	Lemon	Not detected	816,193.33	–
	Geranylacetone	1,424	RS	Tropical, floral, fresh	not detected	418,298	–
Phenol derivatives	Guaiacol	1,090	MS	Phenolic, spicy, vanilla	Not detected	694755	-
	Methyl salicylate	1,281	RS	Minty, sweet, camphor	408,744 ± 212,970	2.18E6 ± 1.04E6	0.0438
	Eugenol	1,392	MS	Nutmeg, cinnamon	Not detected	195,951.33	–
Nitrogen and sulfur compounds	2-isobutylthiazole	1,067	MS	Woody, tomato-leaf notes	6.06E7 ± 1.99E7	2.22E7 ± 7.85E6	0.0359
	Benzothiazole	1,208	MS	Meaty, cooked, beefy	150,945 ± 59,548	88,841.7 ± 33,318.5	0.1901
	Pentane, 1-nitro	900	MS	Algae	7.83E6 ± 1.89E6	1.96E6 ± 503,511	0.0065
	Benzyl nitrile	1,138	MS	Bitter almonds, spicy, floral	433,907 ± 52,391.4	224,000 ± 58,611.4	0.0098
Oxygenated benzene derivatives	Estragole	1,172	MS	Anise	1.79E6 ± 717,612	1.01E6 ± 673,743	0.2430
	Anethole	1,190	MS	Anise	1.27E6 ± 591,754	804,140 ± 490,141	0.3488

In the aromatic profile of the yellow Malacara tomato, the major components (71.79% of its total volatile fraction) are the volatiles derived from nitrogen and sulfur compounds, mainly by 2-isobutylthiazole. Other volatile metabolites were monoterpenes (12.59%), aldehydes (7.24%), and 3.18% due to anethole (oxygenated benzene derivatives). The rest of the chemical families have a low percentage presence, without exceeding 0.67%. In the Malacara red tomato, the fraction of volatile components is more distributed, increasing the weights in some of them, vs. the derived from nitrogen and sulfur compounds. This remains the majority fraction with 44.49% of the total, followed by carotenoids (19.12%), monoterpenes (12.72%), aldehydes (7.86%), phenol derivatives (5.58%), furans (4.62%) due to the only presence of 2-pentylfuran, oxygenated benzene derivatives (3.30%) and 1.05% corresponding to the only detected alcohol (2-phenylethanol). The rest of the chemical families do not exceed 0.6%, being the group of terpenes with a single detected component (camphor) the one that is least represented in the aromatic fraction of the Malacara red tomato. In addition, for the red tomatoes group, the chemical family of esters is not detected in its volatile fraction.

### Correlations Among Traits

**Figure 2:**
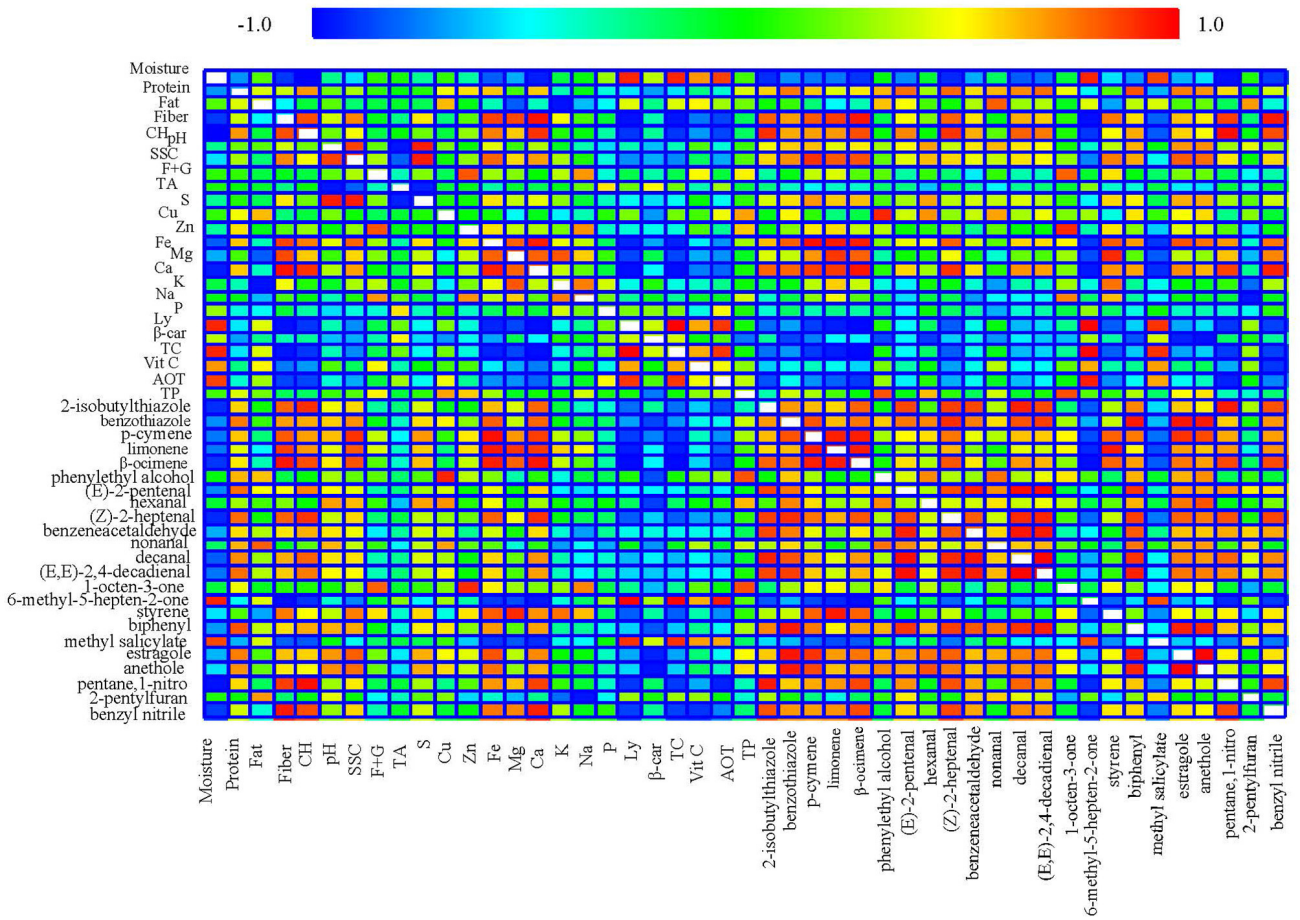
Pearson linear correlations between the traits (nutritional and volatile) studied for the data obtained in the two Malacara tomatoes (yellow and red cv). A positive trend between each pair of variables (red color), a negative trend between each pair of variables (blue color), and no relationship between each pair of variables (green color). The strongest positive/negative relationships are displayed with the greatest intensity of color.

Many significant correlations amongst traits were studied (*n* = 6). Figure 2 represents the Pearson correlations between each pair of variables. These correlation coefficients range from −1 to +1, and they measure the strength of the linear relationship between the variables. Of the correlations detected, standing out are those shown between the most representative molecules involved with carbon (fiber, carbohydrate, soluble solids content) with the minerals [iron (Fe), magnesium (Mg), and calcium (Ca)], and with the more important volatile compounds, mainly of the derivatives of nitrogen and sulfur, terpenes, and (Z)-2-heptenal. The relationships between fiber and carbohydrates, and fiber vs. Fe, Mg, and Ca are statistically significant. Carbohydrates vs. Ca are also statistically significant, and those are related to carbohydrates and fiber and the indicated aromatic components. In addition, these hydrocarbon molecules have negative relationships with the antioxidant parameters analyzed (lycopene and carotenoids, vitamin C, and total antioxidant capacity), showing that these relationships have statistical significance (*p*-values below 0.05). In addition, these hydrocarbon molecules have negative relationships with the antioxidant parameters analyzed (lycopene and carotenoids, vitamin C, and total antioxidant capacity), showing that these relationships have statistical significance (*p*-values below 0.05). In general, the antioxidant parameters (lycopene and carotenoids, vitamin C, and total antioxidant capacity), show negative relationships against the volatile components of Malacara tomatoes, except for 6-methyl-5-hepten-2-one and methyl salicylate, with which positive relationships are found.

The typical aroma of tomato fruits depends on numerous volatile compounds and their synergic effects, the chemical nature, and odor threshold, which can be related to their composition as well crop conditions. In addition, Malacara is a long shelf life type of tomato, whose aromatic components can degrade over time to give rise to other components. Pearson correlations allow obtaining information on the most important relationships and the synergies or antagonisms of the aromatic components of the Malacara tomato and therefore of the possibilities of changes in the evolution of aromatic components in the Malacara tomato. Other significant correlations involved a positive correlation of 2-isobutylthiazole and (E)-2-pentenal, 2-isobutylthiazole and (Z)-2-heptenal, 2-isobutylthiazole and benzeneacetaldehyde, 2-isobutylthiazole and decanal, 2-isobutylthiazole and (E,E)-2,4-decadienal, 2-isobutylthiazole and 1-nitro pentane, 2-isobutylthiazole and benzyl nitrile, benzothiazole and p-cymene, benzothiazole and (Z)-2-heptenal, benzothiazole and decanal, benzothiazole and (E,E)-2,4-decadienal, benzothiazole and biphenyl, benzothiazole and estragole, benzothiazole and anethole, p-cymene and limonene, p-cymene and β-ocimene, p-cymene and estragole, p-cymene and anethole, limonene y β-ocimene, limonene and styrene, β-ocimene and (Z)-2-heptenal, β-ocimene and 1-nitro pentane, β-ocimene and benzyl nitrile, (E)-2-pentenal and (Z)-2-heptenal, (E)-2-pentenal and benzeneacetaldehyde, (E)-2-pentenal and decanal, (E)-2-pentenal and (E,E)-2,4-decadienal, (E)-2-pentenal and biphenyl, hexanal and anethole, (Z)-2-heptenal and benzeneacetaldehyde, (Z)-2-heptenal and decanal, (Z)-2-heptenal and (E,E)-2,4-decadienal, (Z)-2-heptenal and biphenyl, (Z)-2-heptenal y 1-nitro pentane, (Z)-2-heptenal and benzyl nitrile, benzeneacetaldehyde and decanal, benzeneacetaldehyde and (E,E)-2,4-decadienal, benzeneacetaldehyde and biphenyl, decanal and (E,E)-2,4-decadienal, decanal and biphenyl, (E,E)-2,4-decadienal and biphenyl, 6-methyl-5-hepten-2-one and methyl salicylate, biphenyl and estragole, biphenyl and anethole, estragole and anethole, pentane, 1-nitro and benzyl nitrile.

## Discussion

Tomatoes have a very varied carotenoid composition that is able to provide different tonalities from yellow to red. The Malacara tomato is a varietal type included in the group of “for hanging” tomatoes, which are the result of the natural selection carried out historically by farmers, in order to increase the conservation and extend the shelf life of the tomato and improve the quality and adaptation to local edaphoclimatic conditions (35). With this typology, two types differentiated by their color in ripe stage (yellow and red) are studied. Morphologically, Malacara is a tomato with velvety skin, and in terms of external appearance, it could be confused with plum fruit (Figure 1). The sizes found in Malacara tomatoes are within the range indicated by other authors (23, 36, 37). There are “for hanging” tomato lines, with fruit weight ranging from 21.7 to 168.4 g, although large fruits or more than 120 g have little acceptance among farmers and consumers (38), consequently, Malacara tomatoes, regardless of color, may have good commercial acceptance. The number of locules varies from 2 to 3 (Figure 1). This parameter is highly variable among the fruits of “for hanging” tomato lines (37). The spherical shape of the fruits is another attribute that increases the degree of acceptance of the two types of Malacara tomato. The characteristic color of tomato fruits is mainly due to the presence of carotenoids and their composition is influenced by several factors, including geographical origin, fruit maturity, and particularly, the variety (39). Color is one of the most remarkable features among the different tomato fruits and a key factor of external and internal quality and consumer acceptance (40). In fact, it has great importance in evaluating commercials. The color index shows significant differences in the case of both types of Malacara tomatoes. The largest number of “for hanging” tomato lines are slightly intense red fruit colors, there are also pink and orange fruits (41), but as far as in bibliography has been consulted, “for hanging” tomato lines have not been evaluated, of such intense yellow color, as those studied in the present work.

The nutritional composition of long shelf life tomatoes has been studied from different viewpoints. Patanè et al. (42) study the physicochemical components and the activities of enzymes related to the preservation of fruits, of two Sicilian landraces of long-shelf-life tomatoes, to contribute to the diversification in the agri-foods industry production. Conesa et al. (36) collect information on the morphological parameters, pH, soluble solids, and total acidity of Mediterranean landraces in order to highlight the importance of this genetic resource, to evaluate the shelf life of these tomatoes and the potential ability to adapt to changing conditions of the weather. Recently (43), the changes in quality and nutritional traits (dry matter, soluble sugar, organic acids, volatile compounds, and carotenoid contents) of one Italian long-shelf-life tomato landraces, over 120 days of natural storage, have been studied. Mainly to obtain data and to promote to traders and consumers during the winter, and early spring, when high-quality fresh tomatoes are not available. Regardless of these sources, the FAO database (InfoFoods) and the “Pera” tomato (44) were used to establish comparisons with the data obtained. Consequently, Malacara tomatoes have a lower moisture content and a higher energy value. In general, Malacara, mainly the yellow type, is a tomato that doubles the content of protein and fiber and multiplies the carbohydrate content by nine. The fat contents are similar to those shown in the bibliography (44). The quality of tomato concerning mineral contents may vary depending on interactions between cultivars, environmental factors such as light and temperature, the composition of the nutrient solution, crop management practices, and the interaction of all these factors (45). One of the immediate consequences may be that with organic fertilization practices, higher concentrations of potassium accumulate in the edible parts (45), which could explain the high concentrations of potassium in the Malacara tomato, regardless of color.

Regarding fruit quality, soluble solid content in the two types of Malacara tomato studied ranged around 5.87 ± 0.23 °Brix (red tomato) to 6.20 ± 0.20 °Brix (yellow tomato), similar in average to values found by other authors (23, 37, 46) but slightly lower to the values found in Figàs et al. (47) in various landraces of tomato, including some long shelf life varieties. Similarly, when looking at reducing sugars, the mean values obtained for the two types of Malacara tomato are higher than those found by other authors (47) indicating the greater sweet taste of this tomato accession. The average titratable acidity in the two types was also similar to those found for other “for hanging” tomato lines (20, 37, 47, 48). Other authors (46), in Italian growing areas, have obtained means of titratable acidity higher than 1.0% in this type of long-lived tomatoes, possibly due to differences in the ripening stage at the time of harvesting. Regardless of color, in Malacara tomato, the taste index has been higher than 1, which is considered the optimal value for flavor balance (49) in salad tomato, and suggesting that this tomato has an excess of soluble solids. In most *alc* long shelf-life tomato varieties taste index has similar results (37).

The pH values, the soluble solids content, the titratable acidity, and the taste index, the vitamin C content, the antioxidant capacity, and the total phenolic content of Malacara tomatoes are within the range indicated in the literature for other “for hanging” tomato cultivars (23, 35). Compared with the FAO database (InfoFoods) for the “Pera” tomato (44), the yellow Malacara tomato studied contain, on average, around 2-fold more vitamin C and red Malacara tomato around 2.5-fold more vitamin C, and similar content in lycopene.

Lycopene is practically not detected in the yellow Malacara tomato, while the lycopene concentrations in the red Malacara tomato show twice the content of other “for hanging” tomato cultivars (35). Lycopene content and the total carotenoid content are aspects that are related to the pigments and the color that tomatoes develop. In general, ripe long shelf-life tomato fruits do not acquire the typical intense red coloration, with substantially low carotenoid levels. These observations may be related to the *alc* mutation, being able to influence the key regulators of carotenogenesis in the tomato fruits (41), this statement may be the cause of the yellow color in Malacara-type tomatoes.

The perception of flavor based on volatile compounds is very complex, as well as the biochemical chains that intervene in the development of aromas, because volatile compounds are secondary metabolites that, once synthesized, can undergo modifications to produce a new volatile or non-volatile compound. The important volatiles, which positively contribute to tomato aroma, are mainly derived from amino acids (phenolic and branched-chain compounds), fatty acids (includes the most abundant volatiles produced in the tomato fruit), and terpenoids (mono and sesquiterpenoids, and carotenoids). The presence of esters is extremely important to the aroma of fruit in many species. Esters have not been detected in the red Malacara tomato and a small fraction (0.64%) in the yellow tomatoes. Coinciding with these results, green-fruited wild tomato species accumulate considerably higher levels of esters compared with tomato red-fruited, due to the insertion of a retrotransposon in a position adjacent to the most enzymatically active tomato esterase, increasing gene expression. Enzymatic activity results in a dramatic reduction in the levels of many esters that are negatively correlated with human preference (50). In the matrix of Malacara tomatoes, 2-phenylethanol has been detected, which had been previously described as having a positive effect on tomato flavor, increasing floral aroma, and the perception of sweetness (51), this alcohol is 15% more in red tomatoes. Aldehydes, mainly fatty acid derivatives, constitute a class of compounds that include the most abundant volatiles produced in the tomato fruit: the C5 volatiles (E)-2-pentenal, C6 volatiles hexanal, (E)−2-hexenal, or benzaldehyde, C7 volatiles (Z) - 2-heptenal, (E, E)−2,4-heptadienal, and others with a higher number of carbon atoms, like decanal or (E, E)−2,4-decadienal. These compounds are classified as “green leaf” aromas due to their characteristic green, fresh scent of cut grass. In tomato fruits, the production of those compounds is increased at ripening, probably due to the loss of integrity of cellular membranes (52). The long shelf-life characteristic of the Malacara tomato prevents these degradative processes from taking place in the membranes and could be one of the causes of the lower concentration of aldehydes in the Malacara tomato compared with other varieties.

Based on their biosynthetic origin, the volatile compounds carotenoid derivatives are significant in the Malacara red tomato and only 6-methyl-5-hepten-2-one is found in the yellow type. This component is lycopene-derived and is not consistent (53) in its presence in yellow tomatoes. It is possible that the mutation of this tomato causes this concentration, providing an exceptional value for its color and the presence of this carotenoid component. Terpenoids are associated with fresh citrus-like flavors, with warm, peppery notes of the tomato stem, which supplement the aroma and attract consumer attention. Low concentrations of terpenes have been linked to breeding programs primarily focused on larger fruit yields and may have decreased the amount of defensive terpenoids produced in the vegetative part of the plant; therefore, terpene levels are relatively low in tomatoes (53). These compounds are related to numerous roles in plants (they are involved in membrane structure, growth, signaling, and defense mechanisms). The high values of terpenes (~12% in both types of Malacara tomato) may be due to being a native variety. The phenolic volatiles of the Malacara tomato is practically negligible in the yellow type as opposed to the red type. These compounds are involved, positively or negatively, in the human capacity for tomato taste perception, and include a variety of compounds derived from the amino acid phenylalanine and its decarboxylation. Alternatively, it could be transformed into 1-nitro-2-phenylethane or benzyl nitrile by means of other unknown enzymes (54). These enzymatic mechanisms could be responsible for the high concentrations of nitrogen and sulfur compounds in the Malacara tomatoes in yellow and red type, giving rise to more spicy and ripe aromas, compared with floral and fresh aromas.

Although the vitamin C values have been high, our results indicate that in the Malacara tomato, the carotenoids may have a greater contribution to the total antioxidant activity, compared with what was found by other authors (47) where the greatest contribution to the antioxidant capacity comes from ascorbic acid. One of the characteristics of Malacara tomatoes is their low moisture content. The major water content of tomato fruits is negatively related to most of the parameters studied, except with the concentration of lycopene, total carotenoids, vitamin C content, total antioxidant capacity, and the volatile components 6-methyl-5-hepten-2-one and methyl salicylate. It has been shown that carbohydrates, fatty acids present in fat, and amino acids in proteins, represent the natural carbon reserves for the generation of volatile compounds (55). In general with Malacara tomato fruits, the volatile components detected increase when the concentration of the proximate fraction of the tomato is higher. The degradation of carotenoids in the Malacara tomato is responsible for the greater synthesis of the components of the volatile fraction, except in the case of 6-methyl-5-hepten-2-one and methyl salicylate, which are considered volatile derivatives of carotenoids.

Tomato is one of the most consumed and widely grown vegetable crops in the world, but the most commonly stated reason for consumer dissatisfaction with tomatoes is lack of flavor. Factors implicated in the tasteless tomatoes are complex as it comprises several components, including reducing sugars, free acids, minerals, amino acids, and volatile components (56). Factors that are mainly related to the production system, maturity stage at harvest, post-harvest treatment, storage period, and the genotype (57). The feature of the prolonged shelf life of Malacara tomatoes appears to involve attenuation of several metabolic processes associated with slower degradation of cell walls and the sustenance of firmness. In concordance with other “for hanging” tomato cultivars, this trait seems to be correlated with higher sucrose and reduced water loss (41). The high sucrose levels contribute to the Malacara tomato fruit's organoleptic qualities. For this reason, the Malacara tomato in either of its two ecotypes can help to break the trends sensorial of “tasteless tomatoes” and to re-availability of tasty tomatoes, and the results have practical implications for tomato growers, as well as the end consumer.

## Conclusions

Local varieties create the potential to diversify global food production and better enable local adaptation to the diverse and changing environments humans inhabit, offering nutritional and organoleptic diversity. The Malacara tomato is an example of this. In its two versions color, Malacara tomato adjusts to the variability shown of “for hanging” tomato lines, with respect to morphological parameters, providing a yellow color characteristic with high value for commercialization and long shelf-life (12 moths proximally). Although the Malacara tomato is produced during the hot summer months, its long-life characteristic allows it to be consumed fresh, in the winter months, being a high source of bioactive components such as vitamin C, carotenoids, and polyphenols, and an important source mineral, especially potassium, without detracting from its aromatic characteristics.

One of the characteristics of Malacara tomatoes is their low moisture content. The major water content of tomato fruits is negatively related to most of the parameters studied, except with the concentration of lycopene, total carotenoids, vitamin C content, total antioxidant capacity, and the volatile components 6-methyl-5-hepten-2-one and methyl salicylate. In this sense, yellow tomatoes Malacara could be a greater reservoir of proteins, carbohydrates, fiber, and mineral components. While the red tomato Malacara shows a higher concentration of bioactive substances (vitamin C, carotenoids, and antioxidant capacity) and a higher and complex aromatic fraction.

This study provides extensive information on the typing of two Malacara tomato cultivars, differentiated by their color, grown in organic farming conditions that have never been described in the literature. The results incorporate an added value to the native horticultural heritage in the framework of local genetic resource conservation and can help in the selection of suitable tomato varieties, for a particular production system and the selection of local tomato varieties, of high organoleptic quality (rich in flavor and aromas, and attractive colors), high nutritional value and with characteristics to facilitate the improvement of the shelf life of tomato. Furthermore, consumers' dissatisfaction with taste concerning the tomato might be a reason for switching to specialty tomatoes, such as Malacara tomatoes in either of its two versions.

## Data Availability Statement

The raw data supporting the conclusions of this article will be made available by the authors, without undue reservation.

## Author Contributions

MR planned the study and drafted the manuscript. MR and MG-M supervised the research. OC performed the morphological, agronomic, and chemical properties characterization. OC and MG-M performed the chemical composition characterization. OC, MG-M, and MR curated the data. OC and MR performed the statistical analyses. All authors contributed to the article and approved the submitted version.

## Conflict of Interest

The authors declare that the research was conducted in the absence of any commercial or financial relationships that could be construed as a potential conflict of interest.

## Publisher's Note

All claims expressed in this article are solely those of the authors and do not necessarily represent those of their affiliated organizations, or those of the publisher, the editors and the reviewers. Any product that may be evaluated in this article, or claim that may be made by its manufacturer, is not guaranteed or endorsed by the publisher.
